# Effect of Nickel Levels on Hydrogen Partial Pressure and Methane Production in Methanogens

**DOI:** 10.1371/journal.pone.0168357

**Published:** 2016-12-16

**Authors:** Anna Neubeck, Susanne Sjöberg, Alex Price, Nolwenn Callac, Anna Schnürer

**Affiliations:** 1 Department of Geological Sciences, Stockholm University, Stockholm, Sweden; 2 Department of Physical Sciences, The Open University, Milton Keynes, United Kingdom; 3 Department of Microbiology, BioCenter, Swedish University of Agricultural Sciences, Uppsala, Sweden; Martin-Luther-Universitat Halle-Wittenberg, GERMANY

## Abstract

Hydrogen (H_2_) consumption and methane (CH_4_) production in pure cultures of three different methanogens were investigated during cultivation with 0, 0.2 and 4.21 μM added nickel (Ni). The results showed that the level of dissolved Ni in the anaerobic growth medium did not notably affect CH_4_ production in the cytochrome-free methanogenic species *Methanobacterium bryantii* and *Methanoculleus bourgensis* MAB1, but affected CH_4_ formation rate in the cytochrome-containing *Methanosarcina barkeri grown on H*_*2*_ *and CO*_*2*_. *Methanosarcina barkeri* also had the highest amounts of Ni in its cells, indicating that more Ni is needed by cytochrome-containing than by cytochrome-free methanogenic species. The concentration of Ni affected threshold values of H_2_ partial pressure (*p*H_2_) for all three methanogen species studied, with *M*. *bourgensis* MAB1 reaching *p*H_2_ values as low as 0.1 Pa when Ni was available in amounts used in normal anaerobic growth medium. To our knowledge, this is the lowest *p*H_2_ threshold recorded to date in pure methanogen culture, which suggests that *M*.*bourgensis* MAB1 have a competitive advantage over other species through its ability to grow at low H_2_ concentrations. Our study has implications for research on the H_2_-driven deep subsurface biosphere and biogas reactor performance.

## Introduction

Nickel (Ni) is an essential trace metal for most living organisms and especially for methanogens, which use it as a key metal co-factor in many enzymes involved in different parts of their metabolism or in methanogenesis. One of these Ni-containing enzymes, methyl-coenzyme M reductase (MCR) and its co-factor F_430_, which is responsible for the terminal reaction of methane (CH_4_) formation by methanogens, is unique to methanogens. However, Ni is also used in other enzymes involved in methanogenesis, such as carbon monoxide dehydrogenase (CODH) and acetyl-coenzyme A synthetase (ACS) [1]. Under Ni-limiting conditions, however, obligate hydrogenotrophic methanogens have the ability to use a Ni-free hydrogenase, where the reactive center consists only of Fe [2]. This dependence on Ni is suggested to have been a major controlling factor for CH_4_ levels in the early atmosphere of Earth because of high Ni availability in the early oceans [3]. Konhauser et al. [4,5] even suggest that an archean Ni famine might have been a fundamental reason for the drop in atmospheric methane content and the rise in oxygen during the Great Oxygenation Event (GOE). The need for Ni, as well as other trace metals, by methanogens has recently been recognised in research on different biotechnological applications where methanogens play a critical role, e.g. in waste treatment and biogas production [6,7]. In these systems addition of trace metals, including Ni, has been shown to produce positive effects on methanogenic activity and, as a consequence, on overall process performance. Methanogens are key organisms in the degradation of complex organic matter under anaerobic conditions and high microbial activity, specifically of those methanogens using H_2_, is critical for the process. The level of H_2_ is critical for various metabolic pathways, such as the conversion of organic acids, which is thermodynamically unfavourable under high H_2_ pressures [8]. To further understand methanogenic activity, the Ni requirement of methanogenic species has been studied to some extent in pure culture [2,9–16] and lately also as part of bioenergy/biogas research [6]. Studies on pure culture have been restricted to a few species and have mainly focused on the effect of Ni availability on CH_4_ production/growth rate and production of cell mass. In a recent study, addition of trace metals, including Ni, was suggested to result in lower partial pressure of H_2_ (*p*H_2_), resulting in turn in higher methanogen activity and also methanogen diversity [17]. However, to our knowledge no previous study has addressed the importance of Ni for H_2_ consumption and H_2_ threshold levels in pure cultures of methanogen species.

Methanogens are typically classified into three groups, hydrogenotrophic, methylotrophic and acetoclastic, based on substrate and pathway used [18]. Hydrogenotrophic methanogens typically mainly use H_2_/formate as an electron donor, while methylotrophic methanogens can often use both H_2_ and carbon dioxide (CO_2_), as well as methyl compounds such as methanol and methylamines and carbon monoxide (CO). Acetoclastic methanogens mainly use acetate as an energy source. Hydrogenotrophs and methylotrophs in principle use the same pathway for methanogenesis, but with some differences depending on the substrate used [18]. This difference in substrate presence and pathways could affect the need for Ni as a co-factor, and the Ni requirement could thus vary between species belonging to different groups of microbes. Methanogens can also be categorised into those with cytochromes and those without cytochromes, where cytochrome-containing species have a higher ATP gain and thus require a higher threshold pressure of H_2_ to reduce CO_2_ to CH_4_ [2]. Experiments have shown that the growth yield of cytochrome-containing methanogens is more than twice as high as that of cytochrome-free methanogens allowing methanogens without cytochromes to grow on lower partial pressures of H_2_ that is just sufficient to produce enough ATP [19].

The aim of this study was to investigate the need for Ni in production of CH_4_ and consumption of H_2_ by three different pure cultures of methanogens, all using H_2_ as a substrate but with two being strict hydrogenotrophs (*Methanobacterium bryantii* and *Methanoculleus bourgensis* strain MAB1) and one a methylotroph/acetoclast *(Methanosarcina barkeri*). An additional aim was to determine the Ni content in microbial cells and to relate this to production of CH_4_. Availability of Ni affects growth rate and cell mass, but to our knowledge has not previously been analysed in the context of H_2_/CO_2_ consumption/threshold.

## Materials and Methods

Two different experiments was conducted, both investigating three methanogenic strains: i) *Methanosarcina barkeri* (DSM 800), ii) *Methanobacterium bryantii* (DSM 10113) and iii) *Methanoculleus bourgensis* strain MAB1 [20]. *Methanosarcina barkeri* and *M*. *bryantii* were obtained from the Leibniz Institute DSMZ–German Collection of Microorganisms and Cell Cultures. *Methanoculleus bourgensis* MAB1 was provided by the Department of Microbiology, Swedish University of Agricultural Sciences, Uppsala. In the first experiment, the strains were grown in full growth medium (see section "Culture medium and growth conditions" below). The production of CH_4_ was monitored as a function of time and, at the end of the experiment, the microbial cells were analysed for trace metal content (see “Cell digestion experiment”). In the second experiment, all strains were grown in yeast-free growth medium at various Ni levels; normal concentration (0.2 μM), Ni-enriched (4.21 μM) and without Ni addition (0 μM). In this experiment, CH_4_, H_2_ and CO were analysed as a function of time and the microbial cells were analysed for Ni content at the end of the experiment.

### Culture medium and growth conditions

Prior to cultivation, all bottles, black rubber stoppers and plastic disposables used in the experiment were sterilised to remove metal contaminants by soaking in 20% HCl for 24 h followed by soaking in pure Milli-Q water (resisitivity: 18.2 MΩ·cm) for another 24 h. The bottles were then covered with perforated plastic film and dried overnight at 70°C. The Milli-Q water was not treated to strip away Ni and consequently, traces of Ni may be present in the bottles at the onset of the experiments.

The methanogens were grown in bicarbonate buffered basal medium (BM) prepared as described by [21] and modified by [22] by mixing solutions A-I containing (g L^-1^): (A) KH_2_PO_4_, 0.41; (B) Na_2_HPO_4_, 0.43; (F) Na_2_SeO_3_·5H_2_O, 0.3; and Na_2_WO_4_·2H_2_O, 0.3. Solution G was modified by adding (g L^−1^): pyridoxamine, 0.25; nicotinic acid, 0.1; nicotinamide, 0.1; dl-panthothenic acid, 0.05; vitamin B_12_, 0.05; *p*-aminobenzoic acid, 0.05; pyridoxine hydrochloride, 0.1; biotin, 0.02; thioctic acid, 0.05; folic acid, 0.02; riboflavin, 0.05; and thiamine hydrochloride, 0.1. In preparing the medium, 15 mL of solution A, 15 mL of solution B, 1 mL of solution F and 5 mL of solution I were mixed with 1 L of distilled water. The medium was complemented with 0.2 g L^-1^ yeast extract (see “Cell digestion experiment”), boiled down to a final volume of 900 mL (approx. 20 min) and cooled under flushing with N_2_. After cooling, the medium was supplemented with sodium acetate (0.42g L^-1^) and thereafter distributed (18 mL per bottle) into the metal-free bottles under flushing with N_2_. The bottles were closed with the metal-free rubber stoppers (Gotlands gummifabrik) while flushing with N_2_ and sealed with aluminium crimp caps. The bottles were then evacuated to -1 atm pressure and pressurised to 0.2 atm with N_2_ to obtain an oxygen-free environment. This evacuation procedure was repeated three times before the bottles were finally pressurised to 0.2 atm with N_2_-CO_2_ (80:20) and autoclaved for 20 min at 121°C. After cooling, the bottles were supplemented with 1 mL of trace metal mixture C1 (containing 1 mL trace metal solution E, 1 mL vitamin solution G, 12.5 mL solution C and 35.5 mL distilled water to a final volume of 50 mL) and 1 mL of trace metal mixture C2 (containing 49 mL solution D, 1 mL solution H and 0.5 g cysteine-HCl). Mixtures C1 and C2 were prepared separately, sterile-filtered (0.2 μm) into closed autoclaved vials filled with N_2_ and stored cold. The trace metal solution E was slightly modified by using NiCl_2_^.^6H_2_O instead of NiCl_2_ and the amount of CuCl_2_ was adjusted from 0.030 to 0.038 g L^-1^ and MnCl_2_^.^4H_2_O from 0.050 to 0.041 g L^-1^. The concentration of Ni was according to the original (‘normal’) medium for the cell digestion experiment, but adjusted for the second experiment (see below). C1 and C2 were added with a syringe, yielding a final pH of approximately 7.2.

### Cell digestion experiment

The samples of the three methanogenic strains (*M*. *bourgensis* MAB1, *M*. *bryantii*, *M*. *barkeri*) intended for analysis of cell trace metal content were grown in 1.21 L serum bottles by inoculation of 30 mL pre-grown cultures to a total liquid volume of 400 mL reduced BM medium, prepared as described below. After inoculation, the bottles (five per species) were pressured with 0.8 atm H_2_-CO_2_ (80:20). The cultures were grown in the dark at 37°C without shaking. Additional H_2_-CO_2_ (20:80) was added twice during the growth period to a pressure of 1 atm.

The experiment was terminated and cells were harvested in their exponential growth phase. All cultures of the same species were pooled to one batch, in order to increase the cell mass for later analysis. The rate of CH_4_ production was calculated according to the formula for exponential growth, r = (lnN_t_-lnN_0_)/ΔT, where N_t_ is the amount of CH_4_ at the end of the experiment, N_0_ is the amount of CH_4_ at the beginning of the experiment and ΔT is the total time interval.

### Real-Time Quantitative PCR

DNA was extracted using the protocol for Gram-positive bacteria of the DNeasy Blood & Tissue kit (QIAGEN, Germany), with some modifications, i.e. using a starting volume of 500 μL cell suspension, no enzyme added to the lysis buffer, an increased incubation time at 37°C of 45 minutes and a reduced elution volume to 60 μL AL buffer. Both undiluted extracts and a 20-fold dilution of each extract were run by qPCR to detect possible inhibition of the reaction.

Methanogen abundance was measured by qPCR using the CFX96 C1000 Thermal Cycler from Bio-Rad (Hercules, CA, USA). The PCR reaction contained 10 μL iQ SYBR green supermix (Bio-Rad, Hercules, CA, USA), 5 μL nuclease-free water, 3 μL template DNA (1.2–14.6 ng μL^-1^) and 1 μL each of the methanogen-specific primers (10 pmol μL^-1^) Met630F (5′-GGA TTA GAT ACC CSG GTA GT-3′)[1,23] and Met803R (5′-GTT GAR TCC AAT TAA ACC GCA-3) [2,23]. The qPCR programme consisted of a hot start at 95°C for 7 min, followed by 40 cycles of denaturation at 95°C for 40 s, annealing at 60°C for 60 s and elongation at 72°C for 40 s. A melt curve was created at the end of the programme to detect any non-specific amplicons or primer dimers by a temperature rise from 55 to 95°C (ΔT = 0.1°C s^-1^).

An external standard was constructed using the amplicon obtained from the genomic DNA of *M*. *bourgensis* strain MAB1 together with the primers and PCR conditions mentioned above (30 cycles). The purified amplicon (MinElute Gel Extraction kit, QIAGEN, Germany) was then ligated into a vector (pGEM-T easy Vector System I, Promega, WI, USA) and cloned by JM109 High-Efficiency Competent cells. Finally, the plasmids were extracted using QIAprep Spin Miniprep kit (QIAGEN, Germany). The plasmid solution was then diluted in a 10-fold series of dilutions ranging from 1×10^8^ to 1×10^0^ copies μL^-1^ and used for qPCR in the same way as the samples.

The efficiency of the reaction was 103.2% and the correlation factor (r^2^) of the standard curve was 0.999. No inhibition of the PCR was found. The melt curve analysis showed three slightly separated peaks, which were identified as the same band size after a quality check by agarose gel electrophoresis.

The pooled culture liquid was centrifuged using a Beckman J2-HS centrifuge at 15300xg and 10000 rpm for 20 min. The supernatant was then poured off and saved as one sample and the cells were re-suspended in 100 mL wash liquid (basal medium without addition of solutions C1 and C2) and centrifuged. This procedure was repeated one more time. The wash liquid supernatant was also saved as a sample. After the final centrifugation and removal of supernatant, each cell pellet was transferred to a sterile, acid-washed glass flask through suspension in a minimal amount of wash liquid. The weight of glass flask plus wet pellet was noted and the flasks were then dried at 105°C for 48 hours.

The dried cells were digested by wet digestion (modified from Scherer et al. [3,24]) through addition of 2 mL concentrated HNO_3_ to the cell pellet (~100 mg) and boiling for 10 minutes in a sealed acid-washed Duran bottle. Then 2 mL concentrated HClO_4_ (perchloric acid) were added and the sample was heated until white fumes appeared. Next, the sample was diluted with Milli-Q water to a final volume of 10 mL and 0.5 μL concentrated HNO_3_ was added. The mixture was transferred to an acid-washed plastic vial for Inductively Coupled Plasma Atomic Emission Spectroscopy (ICP-AES) analysis. The instrument was calibrated using standard solutions (Multi element standard solutions, LGC-Promochem, chromatography grade) and a blank (Milli-Q water). The analytical error was approximately 4%. The Duran glass bottles used for wet digestion contain 81% SiO_2_, 13% B_2_O_3_, 4% Na_2_O + K_2_O and 2% Al_2_O_3_, which could possibly have influenced the levels of the elements in the cells. The samples (cell pellets, supernatant and wash liquid) were poured into sterile test tubes with 5 μL pure concentrated HNO_3_ (puriss, Sigma Aldrich).

### Ni stress experiments

Differences in growth rate and the need for Ni in *M*. *bourgensis* MAB1, *M*. *bryantii* and *M*. *barkeri* were investigated during cultivation in Ni-limited and Ni-enriched medium. To minimise possible uncontrolled sources of Ni, yeast extract was omitted from the growth medium. The Ni-limited experiments were all conducted in 118 mL borosilicate bottles. The experiments were initiated by inoculation of 10 mL of pure culture, pre-grown without yeast extract and Ni (i.e. Ni was excluded from solution C1), into 20 mL of growth medium containing different amounts of Ni (0, 0.2 and 4.21 μmol L^-1^). The bottles were pressurised to 1 atm H_2_-CO_2_ (80:20) and placed without shaking in the dark at 37°C. One refill of H_2_-CO_2_ (80:20) was made after 119 days of incubation to restore a pressure of 1 atm, when the samples had approximate partial pressure of 0.2 atm. The dissolved element concentrations at the start (the initial 2L batch of growth medium) and end of the experiment were measured in 5 mL aliquots of liquid extracted from the bottles using a sterile needle.

### Analysis

Hydrogen and CO partial pressure (*p*H_2_, *p*CO_2_) were analysed by PP1 (Peak Performer 1, reduced gas analyser) by direct injection of 1 mL withdrawn headspace gas. Methane samples (2 mL) were withdrawn at the same time, injected into glass vials and stored at +2°C until analysis (within a maximum of 5 days) by GC (Perkin Elmer Ariel, Clarus 500 Gas Chromatograph equipped with a TurboMatrix 110 Headspace sampler). Elemental composition of the fluids at the end of the experiments was analysed by inductively coupled plasma atomic emission spectroscopy (ICP-AES) (Spectro, Varian Vista AX with argon as carrier gas). The analytical error was ~4%. All ICP samples were prepared by withdrawing approximately 10 mL centrifuged and filtered liquid and adding 10 μL conc. HNO_3_ (ultrapur, Merck).

## Results

### Trace metals in cells

**Fig 1:**
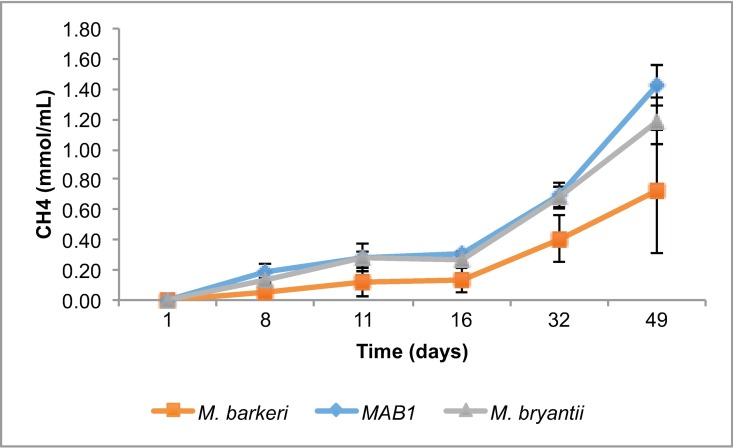
Methane production during growth of Methanosarcina barkeri, Methanoculleus bourgensis MAB1 and Methanobacterium bryantii in a growth medium with added yeast extract.

In cultivation of the different methanogens using standard anaerobic growth medium, also containing yeast extract, *M*. *bourgensis* MAB1 had a slightly higher CH_4_ production rate (0.16% CH_4_ day^-1^), and consequently the highest CH_4_ yield at the time of cell harvest, i.e. after 49 days of incubation (Fig 1). The corresponding CH_4_ production rate by *M*. *barkeri* and *M*. *bryantii* was 0.15% CH_4_ day^-1^ for both species.

Total acquired dried cell mass after 49 days of incubation (pooled cell mass in residual liquid) was 9.40, 12.8 and 9.70 g L^-1^ for *M*. *barkeri*, *M*. *bourgensis* MAB1 and *M*. *bryantii*, respectively. Results obtained in ICP analysis of trace metal concentrations in cells of the different methanogens are presented in Table 1 and S1 Table. Differences in trace metal concentrations between the strains were observed, with *M*. *bryantii* having the highest total metal content per gram of dried cell mass (3.03E+05 ppm g^-1^) and MAB1 the lowest (1.71E+05 ppm g^-1^). Wash liquid analysis showed low levels of metals, indicating a low degree of cell lysis and concomitant leaching of trace metals into the wash medium (S2 Table). Thus, measured trace metal concentrations in the dried cell mass can be considered reliable.

**Table 1:** ICP analysis of iron (Fe), cobalt (Co), molybdenum (Mo) and nickel (Ni) in cell pellets of the three methanogen species investigated. Concentrations are given in ppm g^-1^ of the dried cell material (10 000 ppm = 1wt%). Blank samples are growth medium samples without added microorganisms and no added trace metal solution. DL = detection limit.

	*M*.*barkeri*	*M*. *bourgensis* MAB1	*M*.*bryantii*	Blank (wash medium)
Methane at termination *	526±75.1	1040±99.2	867±113	-
** **	** **	** **	** **	** **
Co**	37.8±1.51	22.05±0.88	12.4±0.5	<DL***
Fe**	671±26.8	821±32.8	1280±51.2	*5*.*53±2*.40***
Mo**	44.5±1.78	55.4±2.22	139±5.58	<DL***
Ni**	38.7±1.55	29.8±1.19	21.6±0.86	*2*.*45±0*.64***
Total content**	**7.92E+02**	**9.28E+02**	**1.45E+03**	*7*.98***

*Total gas content in mmol

**ppm g^-1^ dried cell mass

***μg L^-1^ (see S1 Table for details)

Highest element uptake marked in yellow and lowest in blue.

Iron content in the methanogens ranged from 671 to 1280 ppm g^-1^, with the lowest values observed for *M*. *barkeri* and the highest for *M*. *bryantii*. Among the trace elements, Mo was the most abundant for all three species studied in the cell pellets and Ni the least abundant. Cobalt and Ni were present in similar concentrations in the different species and both Co and Mo were highest in *M*. *bryantii*.

### Impact of Ni on methanogenic growth

**Fig 2:**
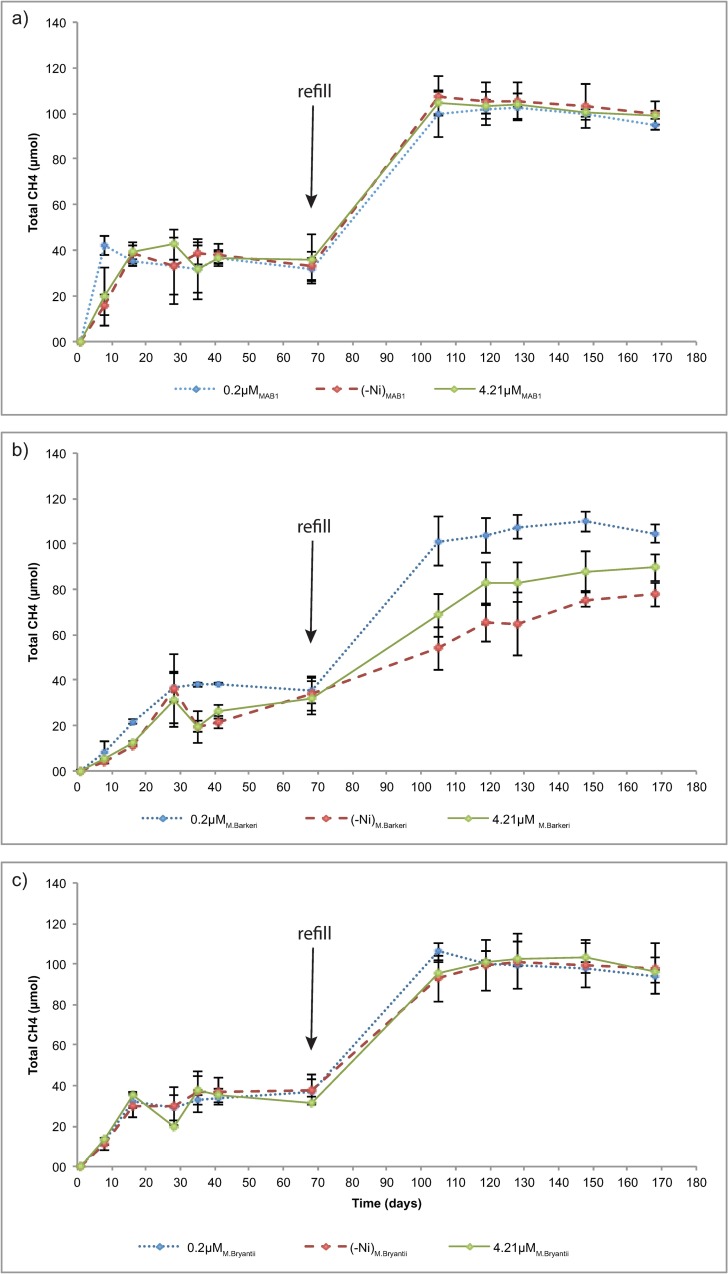
Total CH_4_ formation (μmol) by a) M. bourgensis MAB1, b) M. barkeri and c) M. bryantii, plotted as a function of time. A refill of H_2_/CO_2_ (1 atm) was made before the fifth sampling point (i.e. after 68 days of incubation).

The growth experiments performed at different Ni levels showed that for *Methanoculleus* strain MAB1 and *M*. *bryantii*, neither absence of Ni from the medium nor elevated levels of Ni in the medium influenced the rate or total amount of CH_4_ produced during cultivation (Fig 2 and S3 Table). In contrast, production of CH_4_ by *M*. *barkeri* was clearly reduced both when no Ni was added and when the Ni concentration was increased, with this effect being even more pronounced after a second refill of H_2_ (Fig 2). Total terminal CH_4_ content was highest (104 ± 3.86 μmol) for *M*. *barkeri* when normal medium was used and lowest when excess (89.6 ± 5.94 μmol) or no Ni (77.7 ±5.33 μmol) was added. Analysis of the Ni content in the supernatant growth medium at the end of the experiment confirmed that there were statistically significant differences between the medium supernatants (Table 2).

**Table 2:** Terminal gene abundance (log gene abundance mL^-1^ growth medium) and Ni concentration (μM) in all experiments.

Experiment setup (Ni concentration in μM)	Log gene abundance mL^-1^	±	Terminal Ni (μM)	±
MAB1 (0.2 μM)	8.75	0.86	0.54	22.78
MAB1 (0 μM)	8.78	0.53	0.19	10.75
MAB1 (4.21 μM)	8.59	0.16	3.47	18.6
*M*.*barkeri* (0.2 μM)	8.77	0.4	0.44	11.01
*M*.*barkeri* (0 μM)	9.08	0.61	0.08	1.75
*M*.*barkeri* (4.21 μM)	8.43	0.44	2.77	45.22
*M*.*bryantii* (0.2 μM)	9.06	0.43	0.46	16.7
*M*.*bryantii* (0 μM)	9.05	0.83	0.22	10.14
*M*.*bryantii* (4.21 μM)	9.5	0.81	3.55	52.89

Analysis of the Ni content at the end of the experiment confirmed the differences between species and also showed that the non-supplemented medium had trace amounts of Ni. Quantification of the methanogens at different Ni levels by qPCR at the end of the cultivation experiments showed similar gene abundance (16S rRNA) for all methanogens (Table 2). Acquired value for *M*. *bourgensis* MAB1 was 8.59 ± 0.16 to 8.78 ± 0.53, for *M*. *barkeri* 8.43 ± 0.44 to 9.08 ± 0.61 and for *M*. *bryantii* 9.05 ± 0.83 to 9.50 ± 0.81 log gene abundance mL^-1^ (Table 2). No significant differences in gene abundance between the different species (p = 0.19, F< F crit) at any Ni level (r = -0.04) could be observed using a one-way ANOVA test.

**Fig 3:**
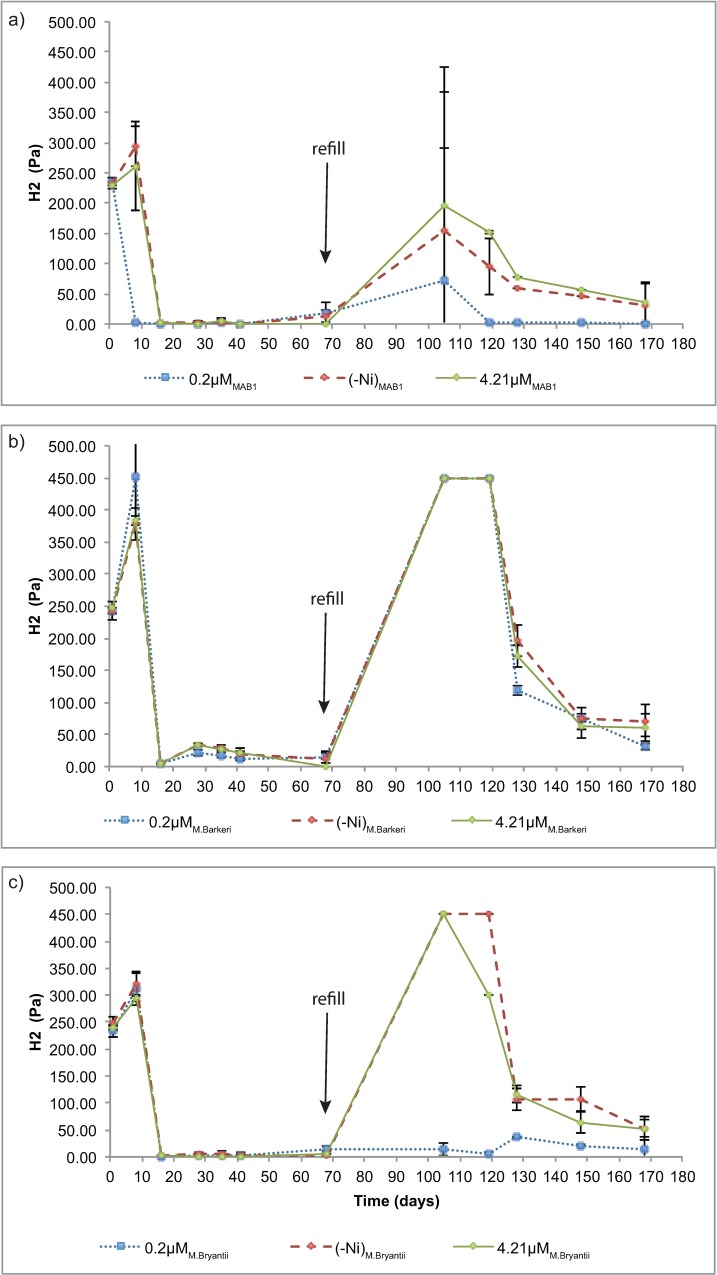
Hydrogen partial pressure (_p_H_2_, Pa) during growth of a) M. bourgensis MAB1, b) M. barkeri and c) M. bryantii, plotted as a function of time. A refill of H_2_/CO_2_ (1 atm, 20:80) was made before the fifth sampling point (i.e. after 68 days of incubation).

Different Ni concentrations in the growth medium had different effects on H_2_ consumption and final H_2_ threshold levels for the three methanogens studied (Fig 3 and S4 Table). For *M*. *barkeri*, no change in H_2_ consumption was seen between the experiments, but a difference was seen for the final H_2_ value, which levelled off at 31.7 ± 5.58 Pa for the full medium, 60.8 ± 21.1 Pa for the high Ni medium and 71.4 ± 24.4 Pa for the medium with no added Ni. The lowest observed *p*H_2_ (13.1 Pa) for *M*. *barkeri* was obtained during growth with normal medium before refilling with H_2_, while the high and no added Ni treatments gave *p*H_2_ values of 22.1 ± 1.41 and 18.6 ± 9.96, respectively. For *M*. *bryantii*, a change in consumption was seen only after refilling with H_2_. Consumption of H_2_ seemed to be faster in cultures grown at standard Ni concentration. As observed for *M*. *barkeri*, the final H_2_ concentration differed in the cultures grown at different Ni levels. In the high Ni medium (4.21 μM Ni), the *p*H_2_ reached 0.87 ± 1.35 Pa and 52.8 ± 22.4 Pa before and after the H_2_ refill, respectively. With the normal medium the final *p*H_2_ value was 14.4 ± 0.33 Pa and for the medium with no added Ni it was 50.6 ± 19.8 Pa. Before the refill, however, the normal and Ni-free treatments reached relatively low *p*H_2_ levels of 3.19 ± 4.63 Pa and 2.50 ± 4.36 Pa, respectively. MAB1 showed a similar trend to *M*. *bryantii*, but the differences in consumption between the different Ni concentrations were less pronounced. Terminal *p*H_2_ after the H_2_ refill was lower for *M*. *bourgensis* MAB1 with the normal growth medium (0.66 ± 0.33 Pa) than with the high Ni medium (36.0 ± 34.2 Pa) or the medium with no added Ni (31.1 ± 35.2 Pa). The *p*H_2_ reached levels as low as 0.10–0.17 Pa in all MAB1 experiments (with or without Ni) before the H_2_ gas refill (Fig 3 and S4 Table).

**Fig 4:**
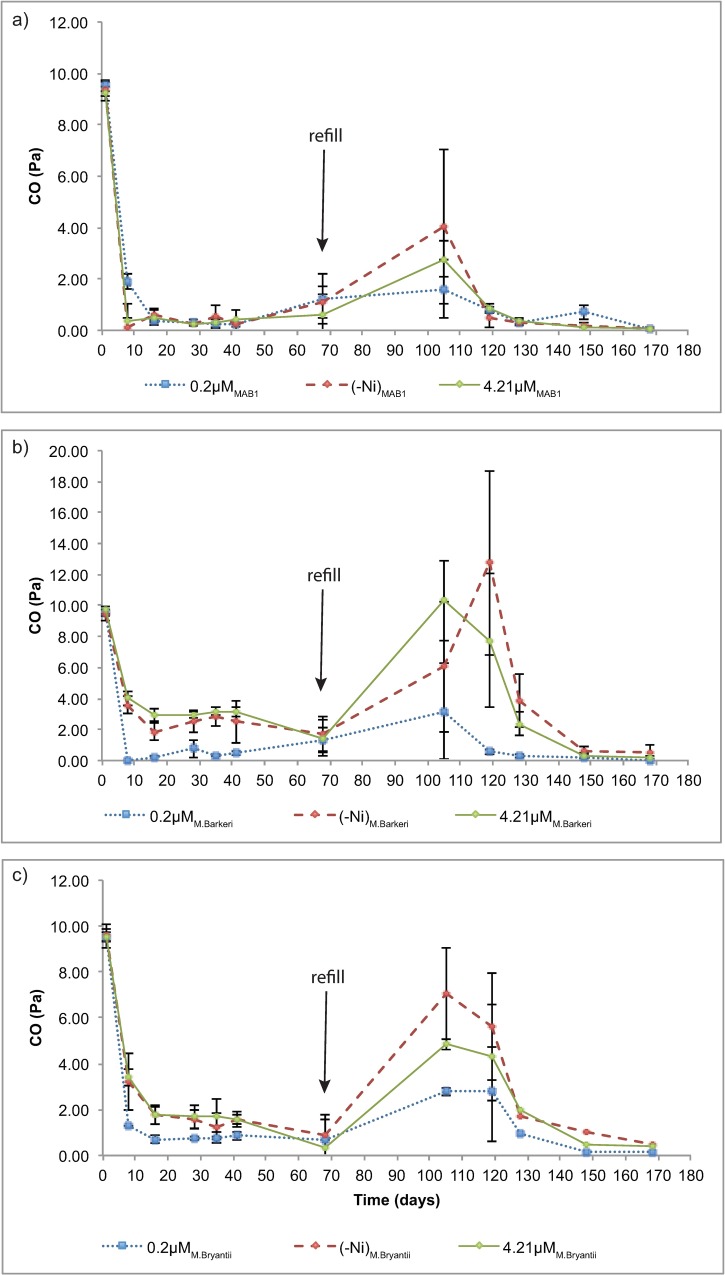
Carbon monoxide partial pressure (_p_CO, Pa) during growth of a) M. bourgensis MAB1, b) M. barkeri and c) M. bryantii, plotted as a function of time.

The level of CO in the cultures decreased over time and no significant differences were observed in the first period between the different media, i.e. with or without added Ni. However, after the H_2_ refill (at 68 days), there was a trend for differences between the no added Ni, Ni-enriched and normal growth medium in the cultures with *M*. *barkeri* and *M*. *bryantii* (Fig 4). Levels of CO were lowest in the cultures with *M*. *bourgensis* MAB1 and its cultures also showed the least difference between the different Ni levels.

## Discussion

### Mo, Co, Ni in cells

The Mo concentration in the cells of the methanogens studied was higher than the Ni and Co concentrations in all three species, but with a relatively higher level in *M*. *bryantii* (Table 1). The opposite was found in a study by Scherer et al. [4,5,24], in which Ni uptake was higher in *M*. *bryantii* than in *M*. *barkeri*. The Ni concentrations in our study (range 22–29 ppm) are low compared with the values of 60–150 ppm reported by Scherer et al. [6,7,24], although the highest Ni content was found in *M*. *barkeri* in both studies. The lower Ni cell content observed here can be attributed to use of 0.2 μM NiCl_2_ growth medium, whereas Scherer et al. [8,24] used growth medium with 5 μM NiCl_2_. In a previous study [2,9–16], growth of a methanogen belonging to the genus *Methanobacterium* (*M*. *thermoautotrophicum*) was stimulated by addition of Ni, Co and Mo, but in contrast to the present study, it was concluded that the requirement for Ni was higher than that for Co and Mo [6,10]. In another study, the growth of *M*. *thermoautotrophicum*, *M*. *bryantii* and *M*. *barkeri* was found to be stimulated by addition of Co and Ni, but not Mo [12,17]. However, that study concluded that growth was best stimulated by addition of all three trace elements together, since with the addition of only one element microbial activity was limited by the requirement for the next most limiting trace element [11]. Growth limitation because of the need for a trace element other than Ni can also explain some of the findings in the present study. The higher concentration of Mo than Ni and Co found in microbial cells is puzzling and could possibly be explained by a difference in the pH of the cells relative to that of the growth medium. Molybdenum is strongly bound to organic compounds and elements at a wide range of pH values and may have accumulated not only in the cells themselves, but also in any organic compounds released into the solution, making Mo more bioavailable [18,25]. Low molecular weight organic compounds are sometimes employed by bacteria and fungi to chelate trace elements, as a way to increase bioavailability, by released chelating agents strongly attached to metal ions and facilitating ion transport into cells [18,26]. However, binding of Ni to organic compounds increases with increasing pH [2,27] and Ni may not be chelated as strongly as Mo. Moreover, compared with Ni, Co is considered moderately bioavailable [19,28] and may thus be less accumulated in cell pellets. Molybdenum concentrations in the wash medium (S2 Table) were below the detection limit, suggesting that a major proportion of the Mo observed originally in the medium (50 μg L^-1^) was actually consumed by the cells.

A positive correlation between Ni and Co was observed for all species, but also similar total concentrations of these two elements in *M*. *barkeri*. The Co present in methanogens is mainly found in corrinoid enzymes and, because *M*. *barkeri* contains the highest amount of corrinoids [20,29], it is logical that this methanogen also had the highest Co concentration (Table 1). The amount of corrinoids are higher in *M*. *barkeri* at methylotrophic than at hydrogenotropic conditions. However, even at hydrogenotrophic conditions, the amount off corrinoids are still higher in *M*.*barkeri* than in *Methanobacterium* species [21,30]. Moreover, it has been shown that the hydrogenotrophic *Methanobacterium* species have a low requirement for Co. In line findings by Florencio et al. [22,29,31], a higher Co content was observed in this study for *M*. *barkeri* and a lower concentration for the hydrogenotrophic *M*. *bryantii*. Methanogens with and without cytochromes sometimes behave differently as regards electron carrier processes and methane yield, and this may also influence trace metal uptake/content in the cells. *Methanoculleus bourgensis* MAB and *M*. *bryantii* are both cytochrome-free species, and thus they have the ability to substitute Fe for Ni (see detailed description below) under Ni-limiting conditions [2]. Nickel limitation most likely prevailed in our experiments, even with the normal medium, due to precipitation of Ni sulphides. In a study by Gonzalez-Gil [11], severe Ni limitation was observed in anaerobic sludge bed experiments, due to precipitation of Ni-incorporating sulphides and, although addition of 4 and 40 μM of Ni increased the CH_4_ production rate during the first 40 hours of growth, it then decreased again. The slow dissolution of sulphides lowered Ni availability in the growth medium and therefore continuous addition of Ni was required in order to overcome precipitation-induced limitation of Ni in the medium. Organic ligands such as EDTA have also been shown to strongly bind to Ni, hindering the uptake of Ni by methanogen cells [32].

### Ni depletion experiment (no added yeast extract)

Previous experiments with a methanogenic pure culture of a non-cytochrome containing hydrogenotrophic methanogen (*Methanobacterium thermoautotrophicum*) have reported slower growth rate during cultivation without Ni, with the maximum growth obtained when 1 μM Ni was added to the growth medium, whereupon growth was limited by the supply of H_2_ rather than the supply of Ni [9]. In the present study, the methanogen species responded differently to the second addition of H_2_ after 68 days of incubation, with divergent CH_4_ formation rates at different Ni levels only for *M*. *barkeri* (Fig 2). This difference in response suggests that the level of Ni rather than H_2_ was restricting CH_4_ formation by this methanogen. Moreover, no significant differences in gene abundance were observed between the different Ni concentrations at the end of the experiments, indicating that all methanogens obtained similar amounts of energy during growth at various Ni levels (Table 2). Formation of CH_4_ is directly coupled to energy production and thus the lowest gene abundance would have been expected for *M*. *barkeri* in the cultures grown without Ni addition, but this was not the case. Instead, the differences in CH_4_ production may be explained by differences in activity and not growth.

Thauer et al. [19] showed that methanogens with cytochromes, such as *M*. *barkeri*, have a higher growth yield than methanogens without cytochromes, such as *M*. *bourgensis* MAB1 and *M*. *bryantii*. Conversion of ADP to ATP in a living cell (standard state at pH 7) requires at least -50 kJ mol^-1^ and conversion of CO_2_ to CH_4_ using H_2_ (ΔG^0^´ = -10 to -40 kJ mol^-1^) allows for synthesis of up to 3 moles of ATP per mole of converted CO_2_. However, this is dependent on *p*H_2_ and, since many hydrogenotrophs are adapted to ATP generation in low *p*H_2_ environments, the growth is lower due to decreased energy gain through reduction of CO_2_. The Ni depletion experiments in the present study confirmed the differences in total CH_4_ production between cytochrome-free and cytochrome-containing methanogens, with *M*. *barkeri* having the highest concentration of CH_4_ throughout the experiment with optimal growth medium (S3 Table). In the experiments with added yeast extract, and in the experiments with non-optimal concentrations of Ni, however, *M*.*bourgensis* MAB1 had an advantage over the other two species.

The importance of yeast extract for CH_4_ production was shown for all three methanogens studied and the total CH_4_ was an order of magnitude higher in the yeast extract-supplemented medium than in the cultures without yeast extract addition. This positive impact of yeast extract is not unique for this experiment or for the species studied but has been described elsewhere, and is most likely an effect of increased activity caused by various growth factors present in the yeast extract [33,34]. The presence of yeast extract also accentuated the difference in CH_4_ production between the three species, suggesting differences in growth requirements.

The results showed that the formation of CH_4_ by *M*. *barkeri* was influenced by Ni limitation, with increased production with increasing Ni concentration in the growth medium (Fig 2). However, the growth rate of *M*. *bryantii* and *M*. *bourgensis* MAB1 did not show any differences between Ni-enriched medium and medium with no added Ni. This seemingly higher need for Ni by *M*. *barkeri* is also supported by its higher levels of Ni per unit dried cell mass (Table 1). Various substrate preferences and metabolic pathways, as well as the fact that *M*. *barkeri* contains cytochromes, could possibly explain these differences in response between the species. For example, *M*. *barkeri* uses a methanophenazine-reducing [NiFe]-hydrogenase (VhtACG), which is restricted to methanogens with cytochromes [2]. In contrast, some methanogens without cytochromes have been shown to synthesise a Ni-free [Fe]-hydrogenase, instead of the F_420_-reducing [NiFe]-hydrogenase, under Ni-limiting conditions, thus reducing their need for Ni. This Ni-free [Fe]-hydrogenase is involved in the reduction of CO_2_ to CH_4_ through reaction 1 [35], and its presence in *M*. *bourgensis* MAB1 and *M*. *bryantii* could explain why these methanogens did not show any reduced CH_4_ production when Ni levels were low (Fig 2). Trace amounts of Ni in the non-supplemented growth medium may also have influenced the outcome and explain the lack of dramatic responsive of the strains to Ni concentrations.

$$Methenyl-H_{4}MPT^{+}+H_{2}\overset{[Fe]-hydrogenase}{\to }Methylene-H_{4}MPT+H^{+}$$ (1)

The level of Ni in the medium not only influenced CH_4_ formation in *M*. *barkeri*, *M*. *bryantii* and *M*. *bourgensis* MAB1, but also somewhat H_2_ consumption and the final *p*H_2_ level, with a pronounced effect after the second refill of H_2_ for all three strains (Fig 3). All three methanogens were efficient in lowering *p*H_2_ during growth in the optimal medium, whereas both Ni-enriched medium and medium with no added Ni seemed to lower the H_2_ consumption efficiency. However, H_2_ uptake in optimal medium with *M*. *bryantii* and *M*. *bourgensis* MAB1 seemed to be most efficient directly after refill, since there was little or no H_2_ peak in those bottles after the H_2_ refill, indicating that H_2_ was rapidly consumed. Excess Ni affected *M*. *barkeri* negatively and *M*. *bryantii* positively before the refill, but by the end of the experiments excess Ni and no added Ni treatments were both equally limiting for *p*H_2_ with all three strains. Addition of no or excess Ni seemed to have no profound effect on H_2_ uptake during a longer time interval.

The toxic concentration of Ni that limits production of CH_4_ has been shown to be 400 μM for an anaerobic methanotrophic-methanogenic granular sludge bed [11] and 60 μM [36] for an anaerobic acetate-enriched digester, but in our experiments limitation in H_2_ uptake and CH_4_ production occurred already at 4.21 μM. However, it is not entirely appropriate to compare pure cultures and sludge cultures because many factors control toxicity, such as the presence of organic acids or syntrophic systems [37,38]. Few previous studies have investigated H_2_ consumption specifically coupled to Ni in pure culture. The H_2_ thresholds observed in the present study varied for the different methanogen species and were clearly also affected by different Ni concentrations (Fig 3), especially by the end of the experiments. Low availability of Ni due to precipitation of insoluble or almost insoluble Ni complexes [32,39] or due to consumption is most likely the reason for the decreased H_2_ consumption after refilling. The lowest *p*H_2_ was obtained for *M*. *bourgensis* MAB1, for which H_2_ consumption produced values as low as 0.1 Pa (S4 Table, Table 3). Previous studies have reported H_2_ thresholds for various methanogens (grown in medium with added yeast) down to partial pressures as low as 0,7 Pa (cf. Table 3). In biogas reactors, *M bourgensis* has been shown to be commonly associated with syntrophic acetate oxidation (SAO), a reaction dependent on very low *p*H_2_ to be energetically favourable, [40]. The extremely efficient H_2_ consumption shown in the present study may explain this correlation to SAO. Studies evaluating the H_2_ threshold for the obligate H_2_-oxidising methanogen *M*. *bryantii* reported values of 0.4 nM H_2_ and showed that at the thermodynamic threshold (i.e. when the available Gibbs (G) free energy is at the lowest level thought to be needed for growth), H_2_ consumption is not controlled by ΔG for the first step in the methanogenesis pathway, where H_2_ is oxidised and CH_4_ is produced (from H_2_ + CO_2_), but by a separate process where electrons from the oxidation of H_2_ are stored in a cellular, solid electron sink [41,42]. Karadagli et. al. (2006) hypothesize, based on thermodynamic calculations and experiments that this electron sink must be a solid-phase component (with an invariant activity) due to the observed oxidation of H_2_ without production of CH_4_ of starved *M*.*bryantii* cells. In their experiments, they repeatedly attained very low H_2_ thresholds, suggesting separate pathways for H_2_ consumption and CH_4_ production. This has also been indicated in studies of syntrophic fermentation reactions, where it has been shown that only one-third of an ATP-unit is sufficient for the conversion of metabolic energy through the transportation of an ion across the cytoplasmic membrane [43].

**Table 3:** Comparison of H_2_ partial pressure (pH_2_, Pa) threshold levels for methanogen species reported in this and other studies.

Species, Ni concentration	*p*H_2_ (Pa) before refill	stdev	*p*H_2_ (Pa) terminal	stdev	Reference no. in
MAB1 No added Ni	0.15	0.13	31.07	35.19	**this study**
MAB1 4.21 μM	0.10	0.02	35.95	34.18	** **
MAB1 0.2 μM	0.17	0.01	0.66	0.33	
*M*.*bryantii* No added Ni	2.50	4.36	50.58	19.78	
*M*.*bryantii* 4.21 μM	0.87	1.35	52.83	22.37	
*M*.*bryantii* 0.2 μM	3.19	4.63	14.41	0.33	
*M*.*barkeri* No added Ni	18.64	9.96	71.42	24.40	
*M*.*barkeri* 4.21 μM	22.06	1.41	60.83	21.10	
*M*.*barkeri* 0.2 μM	13.12	2.12	31.67	5.58	
*M*. *wolfei*			15	-	[44]
*M*. *formicicum*			4	-	[44]
*M*. *barkeri*			46	-	[44]
*M*.*maripaludis*			9	-	[44]
*M*.*bryantii*			2	-	[45]
*M*.*acetivorans*			20	-	[46]
*M*.*thermophila*			50	-	[46]
*M*. *blatticola*			0.7		[47]

Accordingly, the *p*H_2_ threshold does not need to be directly translated to a minimum amount of *p*H_2_ needed for thermodynamically favourable ATP synthesis. Instead, reduction of CO_2_ to CH_4_ could use reducing capacity from stored electrons in the cell, which could explain the low *p*H_2_ for *M*. *bourgensis* MAB1. *Methanosarcina barkeri* had the highest *p*H_2_ among the methanogens studied. In addition to H_2_, *M*. *barkeri* can use acetate for CH_4_ production and, as mentioned previously, uses enzymes that are not present in the cytochrome-free methanogens [48]. These features have been cited in previous studies as an explanation for the higher *p*H_2_ requirement for conversion of CO_2_ to CH_4_ [2,19,49,50]. The two-step acetoclastic pathway used by *M*. *barkeri* evolved through horizontal gene transfer between bacteria and archaea, an event speculated to have occurred no later than 475 Ma, i.e. Mid-Ordovician [51]. Through evolution to this additional metabolic pathway, involving a major change in mechanism of energy conservation, *M*. *barkeri* is believed to have lost its ability to grow under as low *p*H_2_ as the obligate hydrogenotrophs [2,19,52]. Therefore, it is not surprising that *M*. *barkeri* grew at higher *p*H_2_ than the two other species.

Carbon monoxide has been shown to exist in both aerobic and anaerobic waste degradation but the formation of CO is still poorly understood [53,54] especially in pure culture experiments. Methanogens both consume and produce CO using the carbon monoxide dehydrogenase CODH or acetyl coenzyme A synthase (ACS), thus causing pulses of CO forming in anaerobic reactors. It has also been speculated that CO is formed abiotically from the degradation of various organic substrates [53–55]. The source of CO in our experiments is likely both from formation through degradation of organic precursors and from the transferred inoculate at the beginning of the experiments. The decrease in CO was most pronounced during cultivation of *M*. *bourgensis* MAB1 (Fig 4), but did not seem to be affected by Ni level. In contrast, the CO decrease for the cytochrome-containing *M*. *barkeri* was clearly affected by Ni level. These results are in line with the fact that the Wood-Ljungdahl pathway for reduction of CO to CH_4_ is influenced by Ni depletion in cytochrome-free methanogens, through up-regulation of Ni-free [Fe]-hydrogenase [35]. Consumption of CO in *M*. *barkeri* can therefore be expected to be more sensitive to Ni level than in the other two cytochrome-free species. However, *M*. *bryantii* is also affected by Ni concentration, even though it has no cytochromes, because its CO uptake is regulated by CODH and ACS, where the latter has a Ni centre [1]. This would explain the differences in CO uptake with different Ni concentrations observed for *M*. *bryantii* and *M*. *barkeri*. However, it is still unclear why CO uptake by *M*. *bourgensis* MAB1 did not seem to be affected by varying Ni concentrations and further studies are needed in order to explain those results.

## Conclusions

This study showed that Ni availability for methanogens influenced their consumption of CO and H_2_, but not notably production of CH_4_. Nickel absence and enrichment both affected consumption of CO and H_2_, with a more pronounced effect when the level of Ni was low. It should also be noted that even though the rate decrease in gas production was affected by the Ni level, methanogenesis was high even when no Ni was added to the growth medium (although trace amounts were still available). Threshold *p*H_2_ of all methanogens studied was affected by Ni concentration and very low *p*H_2_ values were measured in the experiments with *M*.*bourgensis* MAB1 at optimal growth conditions.

## Supporting Information

S1 Table Element concentrations (μg L^-1^) in the cell digestion experiments. (PDF)

S2 Table Element (Co, Fe, Mo and Ni) concentrations (μg L^-1^) in wash medium, supernatant and cell pellets in the cell digestion experiments. (PDF)

S3 Table Measured accumulated CH_4_ (μmol) at different incubation times (days). (PDF)

S4 Table Hydrogen partial pressure (pH_2_, Pa) (mean of three replicates) at different incubation times (days). The empty cells for days 105 and 119 represent too high content of H_2_ in order to be measured by PP1.
(PDF)
