# Supplementary material for: Effect of Nickel Levels on Hydrogen Partial Pressure and Methane Production in Methanogens
Source: PLoS One. 2016 Dec 16;11(12):e0168357. doi: 10.1371/journal.pone.0168357 (PMC5161503; doi:10.1371/journal.pone.0168357)
Supplement: S1 Table — (PDF) [file pone.0168357.s001.pdf]

S1_ Table. *Element concentration in the cell digestion experiments.*

Element	*M. barkeri*	*M. barkeri ±*	MAB1	MAB1 ±	*M. bryantii*	*M.bryantii ±*
**Al***	164,50	*6,58*	534,87	*21,39*	263,30	*10,53*
**B***	33,93	*1,36*	25,72	*1,03*	60,42	*2,42*
**Ba***	64,38	*2,58*	144,96	*5,80*	304,64	*12,19*
**Ca***	1901	*76,03*	3960	*158,41*	46567	*1862,68*
**Co***	37,83	*1,51*	22,05	*0,88*	12,39	*0,50*
**Cr***	9,81	*0,39*	2,47	*0,10*	5,17	*0,21*
**Cu***	91,62	*3,66*	127,44	*5,10*	197,53	*7,90*
**Fe***	671,11	*26,84*	820,95	*32,84*	1280	*51,20*
**K***	31183	*1247,33*	23518	*940,73*	35619	*1424,74*
**Mg***	666,20	*26,65*	714,31	*28,57*	936,29	*37,45*
**Mn***	5,03	*0,20*	10,63	*0,43*	58,23	*2,33*
**Mo***	44,52	*1,78*	55,39	*2,22*	139,38	*5,58*
**Na***	40554	*1622,17*	33784	*1351,37*	44278	*1771,13*
**Ni***	38,73	*1,55*	29,77	*1,19*	21,55	*0,86*
**P***	100714	*4028,57*	10054<br>7	*4021,89*	166907	*6676,29*
**Pb***	5,32	*0,21*	6,35	*0,25*	8,61	*0,34*
**Rb***	10,57	*0,42*	7,93	*0,32*	10,69	*0,43*
**S***	9375	*375,01*	6152	*246,10*	5687	*227,46*
**Si***	247,33	*9,89*	151,13	*6,05*	270,62	*10,82*
**Sr***	2,21	*0,09*	4,18	*0,17*	19,44	*0,78*
**Ti***	9,91	*0,40*	11,90	*0,48*	10,94	*0,44*
**Zn***	524,31	*20,97*	559,97	*22,40*	802,99	*32,12*
**Zr***	3,51	*0,14*	12,03	*0,48*	-	-
**Total content***	**1,86E+05**	***7,45E+03***	**1,71E<br>+05**	***6,85E+03***	**3,03E+05**	***1,21E+04***

**= ppm of dried cell mass (g)*

Highest elemental uptake marked in yellow and lowest in blue
