# Supplementary material for: Effect of Nickel Levels on Hydrogen Partial Pressure and Methane Production in Methanogens
Source: PLoS One. 2016 Dec 16;11(12):e0168357. doi: 10.1371/journal.pone.0168357 (PMC5161503; doi:10.1371/journal.pone.0168357)
Supplement: S2 Table — (PDF) [file pone.0168357.s002.pdf]

S2_Table. *Cell digestion experiments. Element (Co, Fe, Mo and Ni) concentrations in washing medium, supernatant and cell pellets in µg/L.*

Description	**Co**	**Fe**	**Mo**	**Ni**
Supernatant growth medium *M.bryantii*	2,35	253	4,35	10,1
Supernatant growth medium *M.barkeri*	<DL	8,56	10,8	5,46
Supernatant growth medium MAB1	<DL	41,3	8,62	7,61
Pure basal medium (washing liquid, blank)	<DL	5,53	<DL	2,45
Pure growth medium (with C1 & C2)	50,0	2000	50,0	80,0
The sample, cells dissolved in washing liquid, *M.bryantii*	355	630	418	363
The sample, cells dissolved in washing liquid, *M.barkeri*	282	10500	709	381
The sample, cells dissolved in washing liquid, MAB1	120	12400	1350	209
