# Supplementary material for: Effect of Nickel Levels on Hydrogen Partial Pressure and Methane Production in Methanogens
Source: PLoS One. 2016 Dec 16;11(12):e0168357. doi: 10.1371/journal.pone.0168357 (PMC5161503; doi:10.1371/journal.pone.0168357)
Supplement: S3 Table — (PDF) [file pone.0168357.s003.pdf]

S3_Table. *Measured accumulated CH_4 (μmol) at different incubation times (days).*

Experiment conditions	days of incubation											
	1	8	16	28	35	41	68	105	119	128	148	168
MAB1 0.2μM	0.10 ± 0.05	41.9 ± 4.21	35.5 ± 2.39	32.8 ± 16.5	31.6 ± 13.4	36.8 ± 2.45	31,5 ± 4.94	99,9 ± 10.2	102 ± 7.27	103 ± 6.01	99.7 ± 2.41	95,1 ± 2.23
M.Bryantii 0.2μM	0.21 ± 0.05	12.3 ± 1.65	32.3 ± 3.63	29.1 ± 6.40	32.8 ± 2.20	33.9 ± 2.20	37,1 ± 6.07	106 ± 4.27	100 ± 1.44	99,2 ± 0.41	98,2 ± 2.65	94,0 ± 3.30
M.Barkeri 0.2μM	0.16 ± 0.13	8.15 ± 5.15	21.7 ± 0.89	37.0 ± 6.63	38.0 ± 0.98	38.5 ± 0.37	35,4 ± 5.80	101 ± 10.9	104 ± 7.83	107 ± 5.27	110 ± 4.29	104 ± 3.86
MAB1-Ni	0.08 ± 0.00	16.0 ± 4.64	38.9 ± 4.76	33.2 ± 12.7	38.5 ± 4.92	38.0 ± 4.97	33,1 ± 6.01	108 ± 8.54	105 ± 8.10	105 ± 8.08	103 ± 9.70	100 ± 4.91
MAB1 4.21μM	0.08 ± 0.00	19.7 ± 12.7	39.2 ± 3.06	42.6 ± 6.80	31.9 ± 10.4	36.9 ± 2.88	36,2 ± 11.0	105 ± 5.06	103 ± 3.03	104 ± 1.35	100 ± 1.75	99,3 ± 1.83
M.Barkeri-Ni	0.08 ± 0.00	4.06 ± 0.81	11.0 ± 0.39	36.3 ± 15.3	19.4 ± 2.39	21.6 ± 2.80	34,2 ± 7.60	54.0 ± 9.46	65,2 ± 8.26	64,6 ± 13.7	75,5 ± 3.38	77,7 ± 5.33
M.Barkeri 4.21μM	0.08 ± 0.00	5.16 ± 0.24	12.1 ± 0.81	31.2 ± 11.8	19.2 ± 7.19	26.0 ± 2.90	32,2 ± 7.34	68,5 ± 9.47	82,6 ± 9.62	82,9 ± 8.71	87,8 ± 8.68	89,6 ± 5.94
M.Bryantii-Ni	0.17 ± 0.17	11.0 ± 2.92	30.1 ± 5.91	29.9 ± 9.46	36.8 ± 9.94	37.2 ± 6.94	37,8 ± 7.47	92,8 ± 11.7	99,4 ± 12.7	101 ± 13.5	99,4 ± 11.0	97,6 ± 12.5
M.Bryantii 4.21μM	0.08 ± 0.00	13.5 ± 0.76	35.5 ± 1.63	19.5 ± 3.41	37.6 ± 6.70	35.4 ± 3.26	31,7 ± 2.69	95,8 ± 5.14	101 ± 5.65	103 ± 8.00	103 ± 8.13	96,3 ± 6.63
