# Supplementary material for: Effect of Nickel Levels on Hydrogen Partial Pressure and Methane Production in Methanogens
Source: PLoS One. 2016 Dec 16;11(12):e0168357. doi: 10.1371/journal.pone.0168357 (PMC5161503; doi:10.1371/journal.pone.0168357)
Supplement: S4 Table — The empty cells for days 105 and 119 represent too high content of H2 in order to be measured by PP1. (PDF) [file pone.0168357.s004.pdf]

S4_Table. *The average (three replicates) pH$_2$ (Pa) at different incubation times (days). The empty cells at days 105 and 119 represent too high content of H$_2$ in order to be measured by the PP1.*

Experiment conditions	days of incubation											
	1	8	16	28	35	41	68	105	119	128	148	168
MAB1 0.2µM	235 ± 6.60	1,08 ± 0.54	1,02 ± 1.00	0,61 ± 0.66	1,33 ± 2.07	0,17 ± 0.01	18,81 ± 17.8	24,12 ± 41.8	1,61 ± 0.32	1,91 ± 1.61	1,65 ± 1.03	0,66 ± 0.33
M.Bryantii 0.2µM	233 ± 10.5	311 ± 29.2	1,19 ± 1.11	2,92 ± 3.10	2,42 ± 3.63	3,19 ± 4.63	13,4 ± 6.90	13,29 ± 11.3	4,96 ± 4.05	36,24 ± 1.06	20,91 ± 0.59	14,4 ± 24.3
M.Barkeri 0.2µM	243 ± 4.19	451 ± 85.7	3,75 ± 0,24	21,5 ± 4.41	17,7 ± 3.73	13,1 ± 2.12	14,71 ± 8.99	-	-	119 ± 8.13	76.2 ± 5.92	31,7 ± 5.58
MAB1-Ni	233 ± 10.1	294 ± 32.5	1,16 ± 1.24	1,36 ± 2.44	2,45 ± 5.44	0,15 ± 0.13	12,47 ± 9.35	5,23 ± 10.3	95,13 ± 47.0	59,44 ± 0.36	45,43 ± 0.30	31,1 ± 35.2
MAB1 4.21µM	229 ± 5.72	261 ± 73.5	1,57 ± 1.33	0,38 ± 0.34	4,52 ± 6.39	0,1 ± 0.02	0,25 ± 0.34	76.3	151 ± 1.96	77,9 ± 0.04	55.6 ± 0.02	35,9 ± 34.2
M.Barkeri-Ni	242 ± 13.8	378 ± 25.0	4,88 ± 0.44	33,3 ± 2.32	29.0 ± 4.30	18,6 ± 9.96	10,9 ± 10.9	-	-	196 ± 24.82	74,2 ± 17.3	71,4 ± 24.4
M.Barkeri 4.21µM	248 ± 9.73	384 ± 7.77	4,9 ± 0.16	32,8 ± 2.33	26,5 ± 1.32	22,1 ± 1.41	0,16 ± 0.13	-	-	172 ± 16.58	63,3 ± 18.8	60,8 ± 21.1
M.Bryantii-Ni	248 ± 11.9	321 ± 22.3	3,08 ± 0.58	4,1 ± 4.44	6,13 ± 5.34	2,5 ± 4.36	1,47 ± 2.93	-	-	106 ± 20.0	107 ± 21.8	50,6 ± 19.8
M.Bryantii 4.21µM	240 ± 5.75	294 ± 6.53	2,02 ± 0.78	0,4 ± 0.24	0,51 ± 0.16	0,87 ± 1.35	6,09 ± 5.82	-	-	117 ± 16.8	64,7 ± 20.2	52,8 ± 22.4
